# Antibody-Driven Assembly of Plasmonic Core–Satellites to Increase the Sensitivity of a SERS Vertical Flow Immunoassay

**DOI:** 10.1021/acssensors.4c01052

**Published:** 2024-06-24

**Authors:** Eunice Ebbah, Anthony Amissah, Jun-Hyun Kim, Jeremy D. Driskell

**Affiliations:** Department of Chemistry, Illinois State University, Normal, Illinois 61790, United States

**Keywords:** vertical flow, immunoassay, plasmonic coupling, surface-enhanced Raman spectroscopy, point-of-care, core−satellite

## Abstract

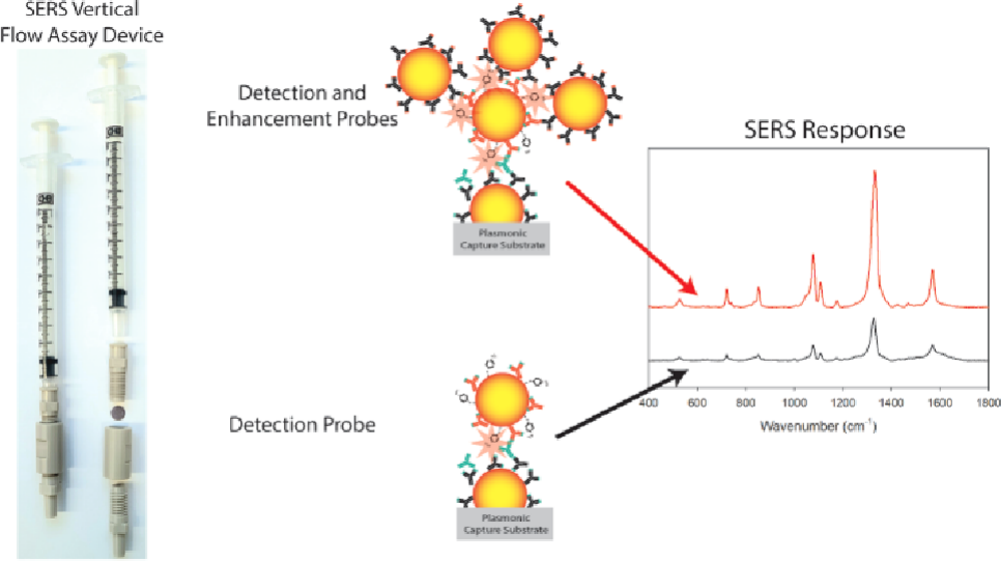

Here, we describe a SERS-based vertical flow assay as a platform technology suitable for point-of-care (POC) diagnostic testing. A capture substrate is constructed from filter paper embedded with spherical gold nanoparticles (AuNPs) and functionalized with an appropriate capture antibody. The capture substrate is loaded into a filtration device and connected to a syringe to rapidly and repeatedly pass the sample through the sensor for efficient antigen binding. The antigen is then labeled with a SERS-active detection probe. We show that only a few Raman reporter molecules, exclusively located adjacent to the plasmonic capture substrate, generate detectible signals. To maximize the signal from underutilized Raman reporter molecules, we employ a secondary signal enhancing probe that undergoes antibody-directed assembly to form plasmonic core–satellites. This facile enhancement step provides a 3.5-fold increase in the signal and a detection limit of 0.23 ng/mL (1.6 pM) for human IgG. This work highlights the potential to rationally design plasmonic architectures using widely available and reproducible spherical AuNPs to achieve large SERS enhancements for highly sensitive POC diagnostics.

Diagnostic tools are critical to the effective management of infectious diseases and population health. Point-of-care (POC) diagnostics, such as rapid antigen tests, play a central role by expanding access to quick results outside the clinic, thereby expediting quarantine or treatment decisions and limiting transmission.^1,2^ Most commercial rapid antigen tests utilize a lateral flow format to generate results in 15–20 min; however, lateral flow assays provide poor clinical sensitivity when benchmarked to reverse transcription polymerase chain reaction (RT-PCR), the standard reference diagnostic test. For example, a review of peer-reviewed clinical data found a pooled sensitivity of 81% for symptomatic patients using commercial COVID 19 lateral flow assays, and the sensitivity fell to 54% when testing 5 days after the onset of symptoms leading to a premature exit from quarantine.^1^ Lateral flow assays achieved only 21% sensitivity for asymptomatic patients who tested positive via RT-PCR. These results highlight the relatively poor clinical accuracy of the currently available POC tests and have driven efforts to develop more sensitive test methods.

Surface-enhance Raman spectroscopy (SERS) is one such readout technology with the potential to meet the demands of POC diagnostics.^3−12^ SERS data acquisition is rapid and can provide the requisite sensitivity with a portable instrument design. Moreover, SERS can facilitate multiplexed detection to screen panels of likely infectious agents based on clinical symptoms. SERS-based assays were first developed using spherical gold nanoparticles (AuNPs) as the plasmonic detection probe.^13^ Spherical AuNPs are stable, reproducibly synthesized with tunable properties, and commercially available, making them a mainstay in SERS and plasmonic-enabled technologies. However, a spherical shape of AuNPs is not optimal for maximizing the SERS signal. To amplify the SERS signal, highly enhancing plasmonic particles have been explored as labels, such as anisotropic and core–shell constructs.^14−16^ The benefit of enhanced signal afforded by these advanced plasmonic particles is often offset by more complex and less reproducible synthesis, making it difficult to standardize across research laboratories and challenging to commercialize the technologies. Rationally designed SERS assays that leverage plasmonic coupling can achieve large enhancement factors for high sensitivity detection, while capitalizing on the attributes of spherical AuNP.^17,18^ Recently, we developed a SERS-based vertical flow assay using antibody-functionalized plasmonic paper as a capture substrate to facilitate plasmonic coupling with the detection probe (Figure S1).^19^ Unlike lateral flow assays, vertical flow assays facilitate rapid immunoreaction between the analyte and label on the capture substrate, where the results are immediately available without the need for assay development time. In addition, the sequential assay procedure prevents the hook effect and provides quantitatively accurate analysis.^20,21^ Sensitive detection was achieved from the Raman report molecules located in the gap between the plasmonic detection probe and underlying plasmonic paper (Figure 1A).^19^ The majority of reporter molecules were not fully located in the “hot spot” and therefore contributed minimally to the detected signal. Thus, there is an opportunity to make more efficient use of Raman reporter molecules. Here, we employ a secondary plasmonic bioconjugate to facilitate antibody-driven assembly of core–satellite structures in combination with our syringe-based vertical flow assay (Figure 1B and Figure S1). This directed assembly forms additional nanogaps to facilitate plasmonic coupling, ultimately leading to more Raman reporter molecules located in “hot spots”. Importantly, this design leverages robust, reproducible, and cost-effective spherical AuNPs.

**Figure 1:**
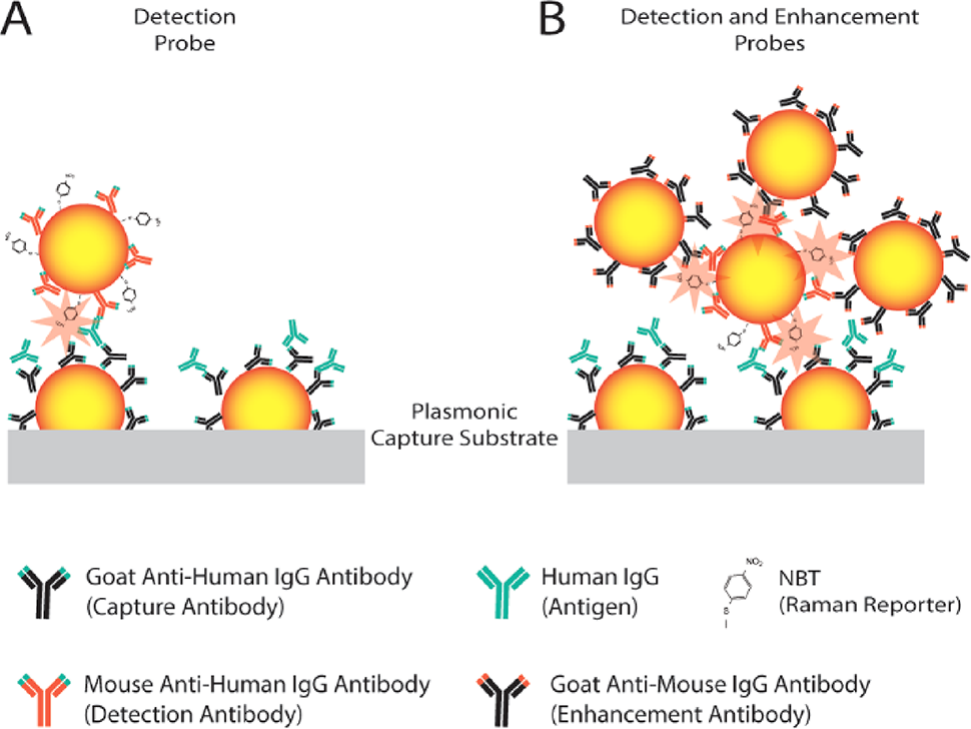
Illustration highlighting SERS hot spots and Raman reporter molecules that generate measured signals in a SERS vertical flow assay. SERS assay performed with detection probe (A) and combination of detection and enhancement probes (B).

To demonstrate that the SERS signal is detected only from Raman reporter molecules in the gaps between plasmonic particles and a coupling partner, we prepared four samples to model our SERS-based vertical flow assay (Figure 2A–D). A gold film was used to model the plasmonic paper capture substrate because it supports plasmonic coupling with an immobilized AuNP, and it allows for SEM imaging to normalize the SERS signal with respect to the density of immobilized AuNPs.^22−24^ The first sample is a self-assembled monolayer of NBT on a smooth gold film without a plasmonic AuNP that served as a control (Figure 2A). Human IgG (hIgG) was spontaneously adsorbed onto a smooth gold film to facilitate the binding of bioconjugates consisting of 60 nm spherical AuNPs functionalized with goat anti-hIgG antibodies (Figure 2B–D). The Raman reporter molecule was co-immobilized with the protein on the AuNP (Figure 2B), the Au film (Figure 2C), or both the Au film and AuNP (Figure 2D). As anticipated, no SERS signal was detected from the Au film-NBT sample without a plasmonic particle, while the three assemblies with AuNPs immobilized above the Au film all resulted in SERS spectra characteristic of NBT (Figure 2E). SEM images of the samples were acquired to quantify the surface densities of immobilized AuNPs (Figure S2). Subsequently, the SERS intensity of the symmetric nitro stretch (e.g., 1338 cm^–1^) was normalized to the immobilized AuNP density to calculate and directly compare the signal generated per AuNP (Figure 2F). Given that NBT on a smooth Au film does not generate an SERS signal, only the NBT molecules on the Au film located directly below the immobilized AuNP-Ab conjugate (Figure 2C) produce a measurable signal. The sample illustrated in Figure 2B yields an equivalent signal to that of Figure 2C, thus confirming that the additional NBT molecules adsorbed onto the AuNP outside of the “hot spot” do not contribute to the measured SERS response. Co-immobilization of NBT on the AuNP and Au film did not lead to a statistically significant increase in the SERS intensity (illustrated in Figure 2D). The mixed monolayer formed by co-immobilization of NBT with protein on both surfaces, e.g., AuNP and Au film, likely resulted in a similar number of total NBT molecules in the gap as a pure NBT-derived monolayer on one surface. Collectively, these data confirm that many NBT molecules present on the AuNP surface do not contribute to the measured SERS signal.

**Figure 2:**
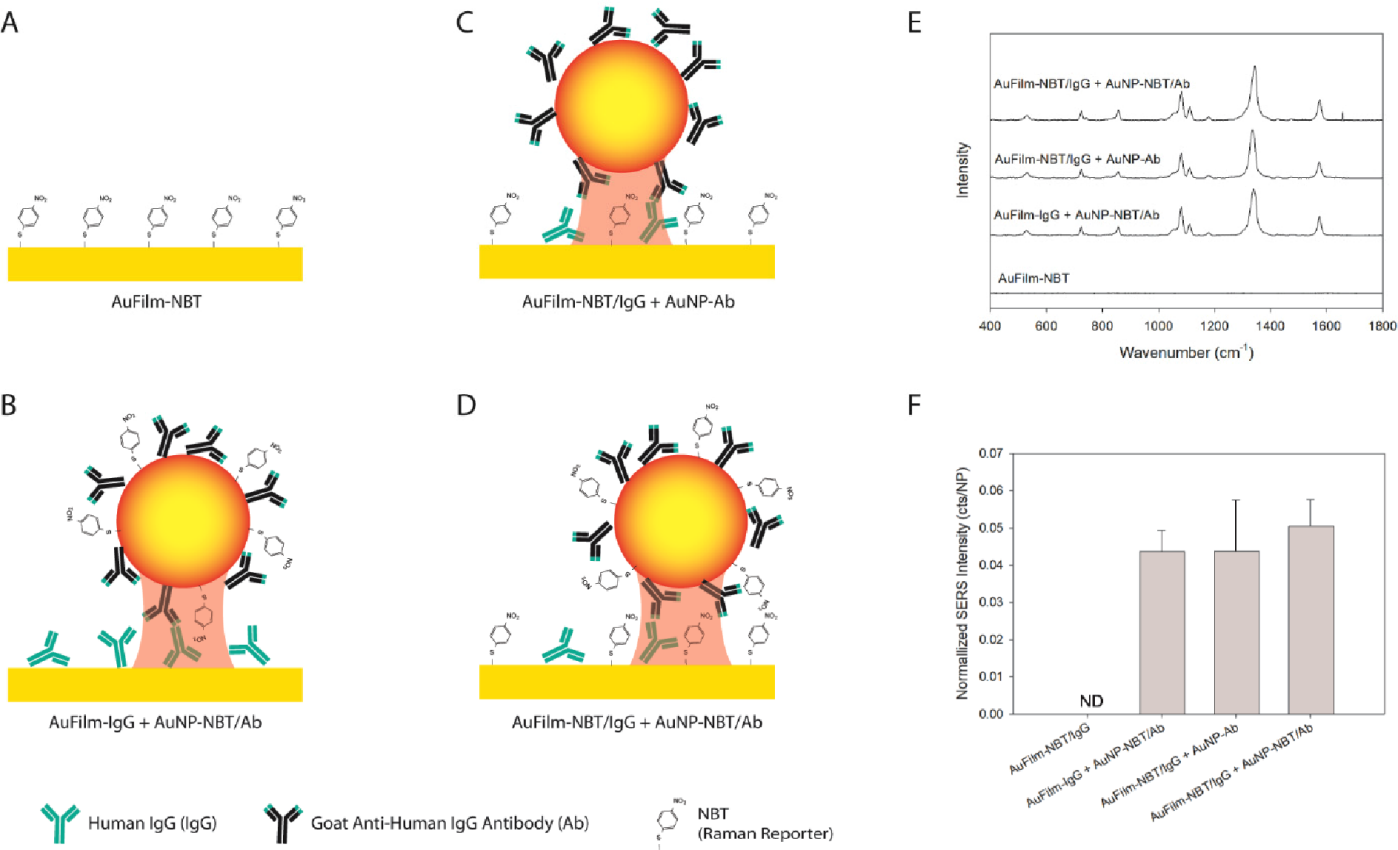
Antibody–antigen mediated assembly of AuNPs on a gold film to experimentally evaluate the location of signal generating Raman reporter molecules in a vertical flow assay. Illustrations representative of samples without a plasmonic AuNP (A) with NBT immobilized on the AuNP (B), with NBT immobilized on the Au film (C), and with NBT immobilized on both the AuNP and Au film (D). Average SERS spectra were collected from two independent preparations of each model system (E). The intensity of the SERS band at 1338 cm^–1^ normalized to the number of AuNPs irradiated by the laser spot, based on SEM imaging (F).

A signal enhancement probe was designed to bind the SERS-active NP label (e.g., detection probe) in the originally developed SERS-based vertical flow assay. This signal enhancement probe serves to form core–satellite assemblies around the detection probe and more effectively use the available but underutilized NBT molecules in the detection probe. To realize the directed assembly of plasmonic particles as conceptualized in Figure 1, the capture and detection antibodies specific for hIgG were derived from different host species, goat and mouse, respectively. The signal enhancement probe was functionalized with goat anti-mouse IgG antibodies to bind mouse IgG and, therefore, selectively form satellites around the detection probes.

Presumptive tests for antibody-directed assembly were performed using antibody-AuNP bioconjugates and aggregation-based assays in suspension.^25^ First, we synthesized goat anti-hIgG AuNP bioconjugates and goat anti-mouse IgG AuNP bioconjugates, representative of the capture antibody and signal enhancing antibody, respectively. These bioconjugates were mixed 1:1, incubated for 1 h to allow for potential binding, and analyzed via dynamic light scattering (DLS) and UV–visible spectrophotometry (Figure S3A and B). The hydrodynamic diameters of the prepared bioconjugates prior to mixing were ∼80 nm, consistent with the adsorption of an IgG monolayer on the 60 nm AuNPs.^26^ The bioconjugate mixture maintained a mean hydrodynamic diameter of ∼80 nm, confirming that the capture and enhancement of antibodies did not interact to form aggregates (Figure S3A). Extinction spectra corroborate the DLS data. The bioconjugates exhibited extinction maxima at 541 nm, a 4 nm red shift relative to the unconjugated AuNPs, and no change in the extinction peak was observed for the bioconjugate mixture (Figure S3B).^27,28^ In the second series of experiments, we synthesized bioconjugates representative of the detection probe and signal enhancing probe, mouse anti-hIgG-AuNP, and goat anti-mouse IgG-AuNP, respectively. Both bioconjugates measured ∼80 nm in diameter; however, a 1:1 mixture of these bioconjugates produced aggregates with a mean hydrodynamic diameter of ∼180 nm (Figure S3C). Moreover, the extinction peak of the mixture shifted to 550 nm and broadened substantially relative to the individual bioconjugates (Figure S3D). The DLS and extinction data confirm aggregate formation and validate the highly selective nature of the goat anti-mouse IgG antibody (e.g., enhancing antibody) toward the mouse anti-hIgG antibody (e.g., detection antibody).

After the antibody specificity was screened, the feasibility of incorporating a signal enhancing probe into the SERS-based vertical flow immunoassay was assessed. Positive control samples, 50 ng/mL human IgG in PBS, or negative control samples, PBS, were passed through plasmonic paper capture substrates functionalized with goat anti-hIgG. Subsequently, the detection probe was infused through the filter paper to label any captured antigen. The assay was completed after this detection probe step for one set of positive and one set of negative samples (i.e., no signal enhancing probe), and the results served to benchmark the impact of an additional signal enhancement step. To another set of positive and negative control samples, a signal enhancing probe (e.g., goat anti-mouse IgG) was passed through the plasmonic capture substrates following the labeling step with a detection probe. The additional signal enhancing step substantially increased the SERS intensity for the 50 ng/mL hIgG positive control sample because of the assembled core–satellite structures (Figure 3), although no visual difference was observed (Figure S4).^17,18^ Importantly, the signal enhancing step did not increase the background SERS signal for the negative control sample, generating an increase in the S/B from 3.3 to 11.8, with and without the enhancing step, respectively. An off-target enhancing probe was prepared using a mouse anti-rabbit antibody to further validate the antibody-directed assembly of the enhancing probe to form core–satellite structures. The anti-rabbit antibody functionalized AuNP is not specific for detection antibody and is expected to pass through the plasmonic filter paper substrate without binding. Figure 3 shows that this off-target enhancing probe did not significantly increase the signal for the 50 ng/mL hIgG sample relative to the assay performed without an enhancing step, confirming the specificity of directed assembly.

**Figure 3:**
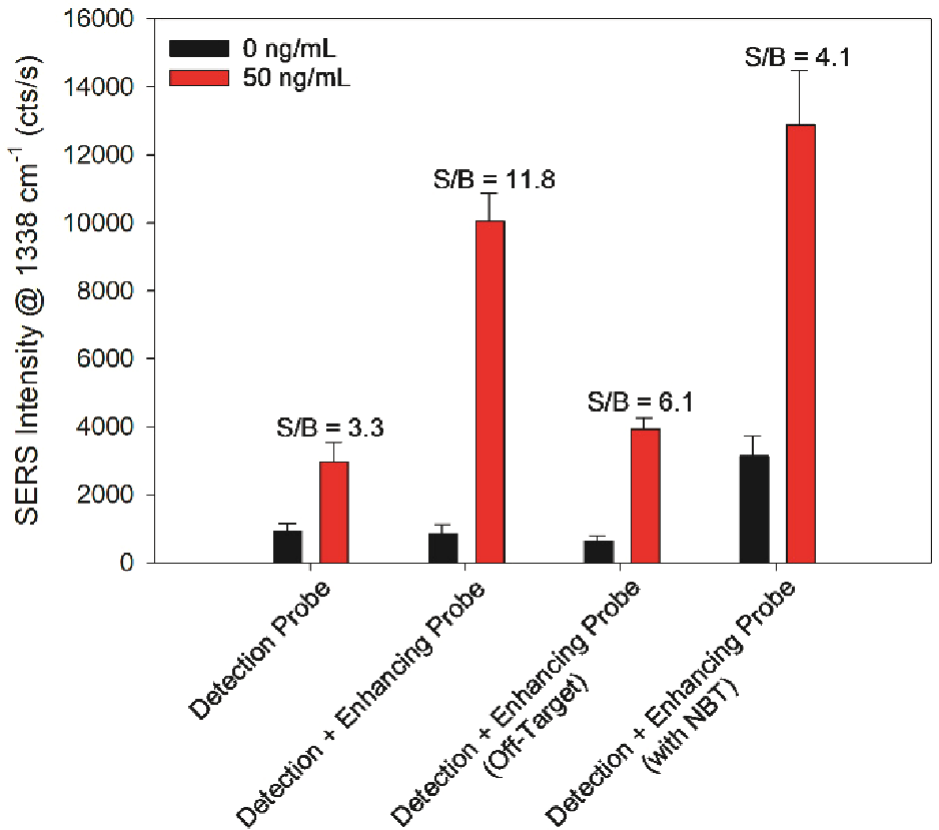
Average SERS intensities for positive (50 ng/mL hIgG) and negative (PBS) control samples analyzed by the SERS vertical flow assay. The assay was performed without the enhancement probe step, with the enhancement probe, with an enhancement probe that is not specific for the mouse anti-hIgG detection antibody, and the specific enhancing probe synthesized with co-immobilized NBT. The ratio of the average signal for the 50 ng/mL sample to the average signal for the 0 ng/mL sample (S/B) is calculated for each assay protocol.

The enhancement probes did not include a Raman reporter molecule (i.e., NBT) and served to amplify the signal of the available Raman reporter molecules located on the detection probe. Based on the results presented in Figure 2F, inclusion of additional NBT on the enhancing probe was not expected to contribute to the detectable signal. Nevertheless, this supposition was experimentally evaluated. A modified enhancement probe was designed to co-immobilize NBT, the Raman reporter molecule, with goat anti-mouse IgG. A slight increase in signal for the 50 ng/mL hIgG positive control sample was observed for the enhancing probe functionalized with NBT; however, a corresponding increase in signal for the negative control was recorded (Figure 3). The NBT-modified enhancing probe is disadvantaged in that nonspecific binding to the plasmonic paper produces a background signal by coupling it to the plasmonic capture substrate. These data suggest that any increase in signal observed for the enhancing probe with NBT was due to nonspecific binding rather than an improved signal from specifically formed core–satellite assemblies. Collectively, these experiments establish that the enhancing probe without a Raman reporter molecule is optimal because it amplifies the analytical signal without contributing to the undesirable background signal regardless of nonspecific binding.

Analytical performance of the SERS vertical flow assay with and without the enhancement step was quantitatively defined by analyzing standard solutions of hIgG prepared in PBS. Each assay was performed in duplicate with independently prepared plasmonic capture substrates to include interassay variability. Figure 4 shows the concentration-dependent response of the SERS intensity for the 1338 cm^–1^ vibrational band. At each concentration, the signal was substantially greater when the signal enhancing step was included. The binding of the enhancement probe increased the signal, on average, by a factor of 3.4 ± 0.4 for each concentration for which a detectable signal was acquired (Table S1). This increase in signal suggests about three to four enhancing probes assembled around each detection probe. This result is consistent with the limit of 60 nm satellites able to surround a 60 nm core based on a rough geometric estimate and is qualitatively supported by the core–satellite hydrodynamic diameters measured in Figure S3C. Regression analysis of the linear region of the calibration curve confirms that the sensitivity is improved by a factor of 3.5 when the signal enhancing step is performed. Moreover, the detection limits, defined as the analyte concentration that generates a signal equal to the blank signal plus 3 times the standard deviation of the blank signal, are 0.23 ng/mL (1.6 pM) and 0.9 ng/mL (6.0 pM) with and without the enhancement step, respectively. These results obtained with highly reproducible and widely available spherical AuNP probes are comparable to SERS-based lateral flow assays reporting 0.1 to 5.0 ng/mL detection limits for IgG that required more sophisticated plasmonic probes to generate large signal enhancements.^7,29^

**Figure 4:**
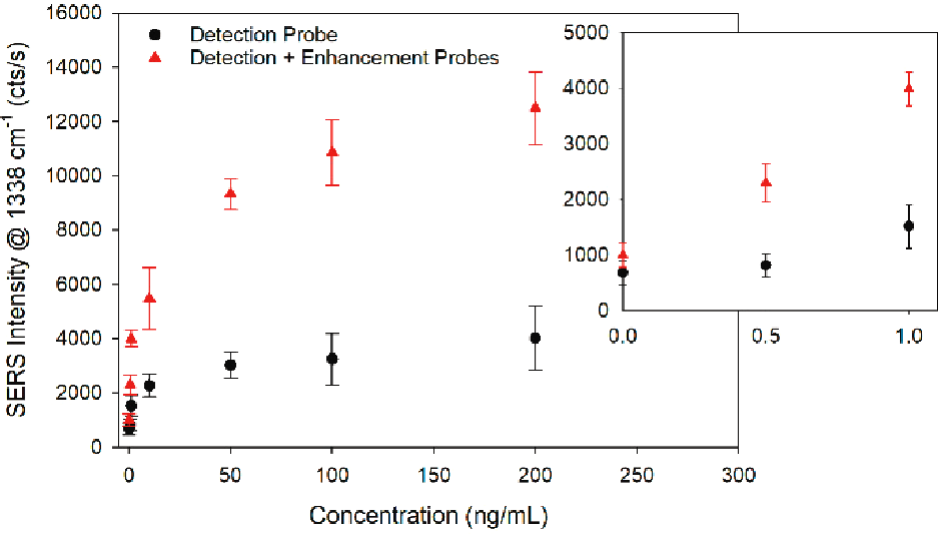
Comparison of SERS vertical flow assay calibration curves for the detection of human IgG performed with and without the signal enhancing probe.

Last, the optimized assay was applied to determine the concentration of hIgG in normal human serum and test the performance of the assay in a complex matrix. IgG is an abundant protein in human serum with a normal concentration range of 8–18 mg/mL. For this analysis, we prepared two separate normal human serum samples and diluted the serum samples to 1:10^6^ using PBS to extend the assay dynamic range. A calibration curve using a set of hIgG standard solutions was generated and is presented in Figure S5. Using the best fit regression to the calibration data and the signal for the diluted serum samples, the IgG concentrations measured 10.3 and 11.4 ng/mL for the two serum samples. Accounting for the dilution factor, the original whole serum samples contained IgG at 10.3 and 11.4 mg/mL, both within the expected normal concentration range of 8–18 mg/mL.^30^

In summary, we developed a paper-based SERS vertical flow immunoassay by leveraging plasmonic coupling to maximize assay sensitivity. Our results highlight the opportunity to more efficiently enhance the signal from underutilized Raman reporter molecules on commonly used SERS detection probes. Specifically, a signal enhancing bioconjugate was developed for the in situ assembly of plasmonic core–satellites directly on the sensing platform. Our approach utilizes spherical AuNPs to circumvent some of the challenges associated with reproducible synthesis of novel plasmonic nanomaterials and issues with nanoparticle instability in a suspension. The optimized assay was implemented for the detection of hIgG, providing a detection limit of 0.2 ng/mL (1.6 pM) and accurately quantifying IgG levels in human serum with a total sample analysis time of approximately 5 min. This sensitive and rapid platform has the potential to improve the clinical sensitivity of point-of-care testing and advance disease management. Moreover, we expect that signal enhancing bioconjugates can be tailored to bind and form satellites around any SERS-active detection probe; thus, this strategy is generally applicable to many SERS-based assays and extends beyond that of the vertical flow format.

## Supporting Information Available

Experimental methods, table of SERS intensities for assay with and without enhancement step; photograph of plasmonic paper and vertical flow device; SEM micrographs of detection probes immobilized on a gold film; DLS and UV–visible extinction spectra of bioconjugates and bioconjugate mixtures; SEM images of plasmonic paper; additional calibration curve for hIgG detection and serum analysis (PDF)
