# Supplementary material for: Antibody-Driven Assembly of Plasmonic Core–Satellites to Increase the Sensitivity of a SERS Vertical Flow Immunoassay
Source: ACS Sens. 2024 Jun 24;9(7):3496–501. doi: 10.1021/acssensors.4c01052 (PMC11287735; doi:10.1021/acssensors.4c01052)
Supplement: Supplementary file 1 — se4c01052_si_001.pdf [file se4c01052_si_001.pdf]

Supporting Information for:

Antibody-Driven Assembly of Plasmonic Core-Satellites to Increase the Sensitivity of a SERS Vertical Flow Immunoassay

Eunice Ebbah,[†] Anthony Amissah,[†] Jun-Hyun Kim,* and Jeremy D. Driskell*

Department of Chemistry, Illinois State University, Normal, IL 61790, USA.

[†]These authors contributed equally to this work.

Corresponding Authors: jkim5@ilstu.edu and jdriske@ilstu.edu

EXPERIMENTAL DETAILS

Materials

Tetrachloroauric (III) acid ($HAuCl_4 \cdot 3H_2O$, 99.99% metal basis) and citric acid trisodium salt dihydrate (99%) from Acros Organics were used to synthesize gold nanoparticles (AuNPs) for the preparation of plasmonic paper. AuNPs with a nominal diameter of 60 nm were purchased from Ted Pella Inc. and used to synthesize the detection probes. Bovine serum albumin lyophilized powder (BSA) was obtained from Sigma Aldrich. 4-nitrobenzenethiol (NBT, 96%), goat anti-human IgG antibody (31119), mouse anti-human IgG antibody (31137), goat anti-mouse IgG antibody (RI 43719), normal human serum, Whatman grade 4 filter paper, phosphate buffered saline (PBS; 10 mM, pH 7.4), and trehalose anhydrous were purchased from Thermo Scientific. IgG from human serum (I4506) was acquired from Sigma-Aldrich. Gold film substrates prepared with a 5.0 nm Ti adhesion layer and 100 nm Au layer deposited via resistive evaporation on a glass microscope slide were purchased from EMF Corporation. A male luer adapter to 1/4-28, 1.5 mm bore, PEEK (JR-CMlAPK), female luer adapter to 1/4-28, 1.5 mm bore, PEEK (JR-CFLAPK), and union, PEEK, LP 1.3 mm bore, body only, 1/4-28 (JR-065) were purchased from Analytics Shop to construct the immunofiltration device.

Preparation of Bioconjugate Probes Immobilized on a Gold Film

Gold-coated microscope slides were cut into 1×1 cm^2 pieces. A subset of gold film substrates was prepared with a monolayer of NBT by submerging in 1 mM NBT (prepared in acetonitrile) overnight, followed by rinsing with ethanol and drying with a stream of nitrogen gas. Additional subsets of gold film substrates were prepared with an adsorbed layer of human IgG (hIgG) or a mixed layer of NBT and hIgG, followed by binding of a goat anti-human IgG-AuNP bioconjugate or SERS active bioconjugate formed with NBT and goat anti-human IgG co-immobilized on AuNPs. An adlayer of human IgG was formed by placing 25 μL of 1 mg/mL human IgG in PBS on each 1×1 cm^2 Au film substrate and incubating for 4 h. This incubation was conducted in a humidity chamber consisting of a parafilm sealed petri dish with water droplets surrounding the substrate to prevent sample evaporation. A mixed adlayer of NBT and hIgG was formed by placing 25 μL of solution containing 1 mM NBT and 1 mg/mL hIgG in PBS on each 1×1 cm^2 Au film substrate and incubating for 4 h in a humidity chamber. Excess hIgG or hIgG/NBT mixtures were removed

by thoroughly rinsing with PBS and 25 μL of an appropriate bioconjugate, which were added to each substrate for a 4 h incubation step in a humidity chamber. The bioconjugates were either AuNP coated with goat anti-human IgG or AuNP coated with a mixture of NBT and goat anti-human IgG, and bioconjugate preparation is detailed below. The substrates were then rinsed with PBS and dried with a stream of nitrogen gas. SERS spectra and SEM images were collected for each sample.

Fabrication and Characterization of Plasmonic Capture Substrate

Spherical AuNPs (60 nm) were synthesized and used in preparing plasmonic papers. AuNPs were synthesized using a thermal reduction method.[1] Highly concentrated AuNPs are required for the fabrication of plasmonic paper and commercial AuNPs at the requisite concentration were cost-prohibitive; thus, AuNPs were synthesized in-house for this purpose. An aliquot (2.0 mL) of gold solution (1 wt% $HAuCl_4 \cdot 3H_2O$) was diluted to 100 mL with nano pure water (18.1 MΩ; Barnstead/Thermolyne Purification system equipped with a 0.2 μm membrane filter) in a 250 mL Erlenmeyer flask. The solution was heated to boiling while vigorously stirring. At the onset of boiling, trisodium citrate (1.5 mL of 1 wt%) was quickly added to the boiling solution and the heat was turned off. The resulting solution was then allowed to stir for 40 mins while cooling to room temperature, resulting in the formation of spherical AuNPs. The final AuNP suspension was highly concentrated by centrifugation and used without further purification to prepare plasmonic filter paper as the capture substrate.

Plasmonic capture substrates for the SERS assay were prepared using Whatman grade 4 filter papers with 20-25 μm pores size (i.e., particle retention), following a previously reported dip coating method.[2] This cellulose-based paper substrate offers a clean SERS background upon the incorporation of plasmonic metal nanoparticles and is an ideal support membrane for a SERS vertical flow assay.[3-5] Briefly, the filter paper was dried at 40 °C overnight in an oven and subsequently submerged in 10 mL of the synthesized AuNP suspension in a plastic petri dish (60 mm x 15 mm). After soaking for 24 h, the filter papers were removed from the AuNP suspension, rinsed with 95% ethanol to remove excess AuNPs, and dried in an oven set to ~40 °C for 30 mins. A paper punch was used to cut 5 mm diameter discs from the resulting plasmonic paper to be utilized for the SERS vertical flow assay.

The AuNP-embedded filter paper was functionalized with capture antibody by applying 2 μL of 1 mg/mL goat anti-human IgG in PBS containing 1% (wt/v) trehalose and drying in an oven for 30 min at 37 °C. The capture antibody spontaneously adsorbs onto the AuNPs, and the trehalose stabilizes the dried antibody. Prior to use in an assay, the capture substrates were blocked with 100 μL, 1% (wt/v) BSA solution for 1 hour to reduce nonspecific binding.

Detection Probe Synthesis

SERS-active detection probes were prepared following a previously established procedure, in which a Raman reporter molecule and a detection antibody were co-adsorbed onto AuNPs.[6,7] It is worth noting that NBT was selected as the Raman reporter molecule because of its inherently strong Raman signal originating from the symmetric nitro stretch. Additionally, AuNPs used in the preparation of the detection probes were purchased from a commercial source that provided better monodispersity and analytical precision than in-house synthesized AuNPs. A 1.0 mL aliquot of 60 nm AuNPs (Ted Pella, Inc.) was added to a microcentrifuge tube and 40 μL of 50 mM phosphate buffer (pH 8.0) was introduced to adjust the pH of the solution. Mouse anti-human IgG antibody (30 μg) and 10 μL of 1 mM 4-NBT were added to the AuNPs. This solution was thoroughly mixed and allowed to rest for 90 min at room temperature, allowing

spontaneous adsorption of the antibody and NBT onto the AuNPs. The suspension was centrifuged at 5000 g for 5 mins and the supernatant was discarded to remove excess unbound antibody and NBT. Afterwards, the ERLs were resuspended in 2 mM phosphate buffer (pH 8.0) and centrifuged followed by resuspension in the buffer for two additional times to thoroughly wash off all unbound antibodies and NBT. BSA (10 µL at 10% wt/v) and sodium chloride (10 µL of 10% (wt/v) NaCl were added to the purified detection probes to block off any unbound surface of the AuNPs and increase the ionic strength, respectively.

Signal Enhancement Probe Synthesis

The synthesis of the signal enhancement probe follows a similar procedure as the preparation of the detection probe, with some slight modifications. A 1.0 mL aliquot of 60 nm AuNPs (Ted Pella, Inc.) was added to a microcentrifuge tube and 40 µL of 50 mM borate buffer (pH 9.0) was introduced to adjust the pH of the solution. Goat anti-mouse IgG antibody (30 µg) was added and incubated for 90 mins to allow maximum adsorption of the antibody onto the AuNPs surface. The bioconjugate suspension was centrifuged at 5000 g for 5 mins and the supernatant was discarded to remove excess antibody that was not bound onto the AuNPs. Afterward, the pellet was resuspended in 2 mM borate buffer (pH 9.0) and centrifuged followed by resuspension in the same buffer two additional times to thoroughly wash off all unbound antibodies. Lastly, 10 µL of 10% (wt/v) BSA and 10 µL of 10% (wt/v) aqueous NaCl were added to the AuNP-Ab signal enhancement probe.

Vertical Flow Assay Protocol

This assay was developed from a previously established procedure with some modifications.[6] Here, a calibration curve was derived by diluting 1 mg/mL hIgG stock solution in PBS into various concentrations (0.5, 1, 10, 50, 100, and 200 ng/mL). PBS was used as a negative control to determine the nonspecific binding of the detection probes. The SERS capture substrate comprising of a BSA-blocked plasmonic paper with pre-immobilized goat anti-hIgG was placed in a syringe filter apparatus and the fittings were screwed to keep the capture substrate in place (**Figure S1**). A 100 µL sample solution containing antigen or PBS negative control was introduced onto the SERS capture substrate by a syringe and cycled through the capture substrate, e.g., plasmonic filter paper modified with capture antibodies, to bind and concentrate the antigen on the sensing surface. Next, 100 µL of detection probe, AuNP modified with NBT and mouse anti-human IgG antibody, was passed through the capture substrate twenty times for optimal interactions and labeling of the captured antigens. Signal enhancing probes, AuNP functionalized with goat anti-mouse IgG antibody, were cycled ten times through the capture substrate to specifically bind to detection probes, thereby forming core-satellite assemblies. Lastly, a 100 µL wash buffer (1% wt/v BSA, PBS and Tween 20) was then cycled through the filter ten times followed by two additional rinses to remove any non-specifically bound components on the plasmonic paper. The combined assay protocol, including rinsing steps, required approximately 5 min to complete. The paper was removed from the filter holder apparatus, dried, and analyzed for SERS response.

Instrumentation

UV-visible Spectrophotometer. The confirm the successful synthesis of the AuNPs and determine the amount of particles loaded on plasmonic papers, UV-Vis was used to measure the extinction of the AuNP

suspension before and after loading onto the filter paper. The UV-visible instrument used for this analysis was an Agilent 8453 spectrophotometer (Agilent Technologies, Santa Clara, CA). Prior to sample analysis the instrument was blanked with nanopure water. Adsorption of antibodies onto AuNPs to form bioconjugates, e.g., detection and signal enhancing probes, was confirmed by shifts in the LSPR band position using a Cary 3500 UV-visible spectrophotometer (Agilent).

Scanning Electron Microscope. SEM was used to visualize and quantify the surface density of bioconjugates bound to a smooth gold film support. Additionally, SEM was used to analyze the successful preparation of plasmonic papers by visualizing the topography and size distribution of the AuNPs on the plasmonic paper. A field emission SEM (Zeiss Sigma 300 VP) equipped with a Gemini column, operating at low voltages was used to acquire images.

Dynamic Light Scattering (DLS). A Malvern Zetasizer Nano ZSP was used to confirm the successful adsorption of the antibodies on the AuNPs to form bioconjugates. This was achieved by measuring and comparing the mean hydrodynamic diameter and polydispersity of unconjugated AuNPs and formed bioconjugates. Unconjugated AuNP and AuNP probes were placed in a micro-volume disposable Eppendorf cuvette for DLS analysis and equilibrated for 60 s at room temperature prior to data acquisition. Each size measurement was determined from 10 runs, 10 s each and reported as Z-average values.

Raman Spectrometer. SERS spectra were collected using an Enwave Optronics, Inc. ProRaman-L-785B instrument configured with a 785 nm excitation source set to 10 mW at the sample surface and a high-sensitivity CCD thermoelectrically cooled to -60 °C. The laser was focused on the sample (Au film substrate or plasmonic paper) placed on an x-y-z sample stage. For the samples prepared on a gold film substrate for interrogating plasmonic coupling, two of each sample type were prepared and 8 spectra were collected from random locations on each sample. For the vertical flow assays, each sample (e.g., standard solution, negative control, or human serum) was analyzed on a minimum of two capture substrates fabricated in independent batches. Five spectra were acquired from each paper at random locations. All SERS spectra were baseline corrected using the auto-baseline algorithm built into the Enwave application software (ProRaman Reader V8.2.8).

Table S1. Corrected Peak Intensities for the Analysis of hIgG Standard Solutions

hIgG Concentration (ng/mL)	$I_{Detection\ Probe}$ (cts/s)	$I_{Detection + Enhancing\ Probe}$ (cts/s)	$I_{Detection + Enhancing\ Probe}/I_{Detection\ Probe}$
**0**	0	0	-
**0.5**	137*	1297	-
**1.0**	842	2984	3.5
**10**	1593	4465	2.8
**50**	2343	8334	3.6
**100**	2566	9851	3.8
**200**	3342	11496	3.4
**Average**			**3.4**
**Std. Dev.**			**0.4**

*Less than the lowest detectable signal

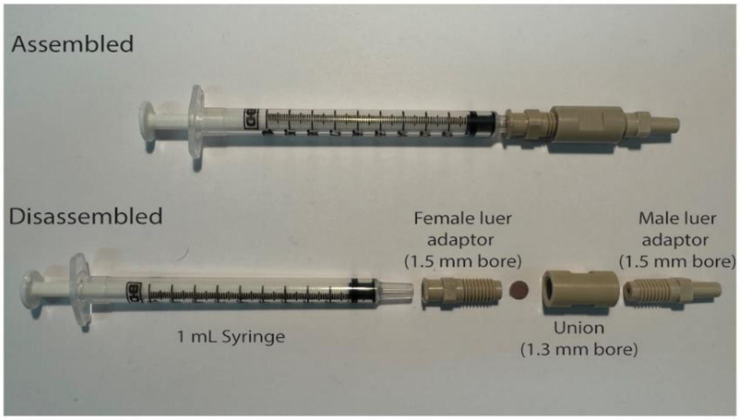

Figure S1. Vertical flow immunoassay device using syringe-based filtration apparatus.

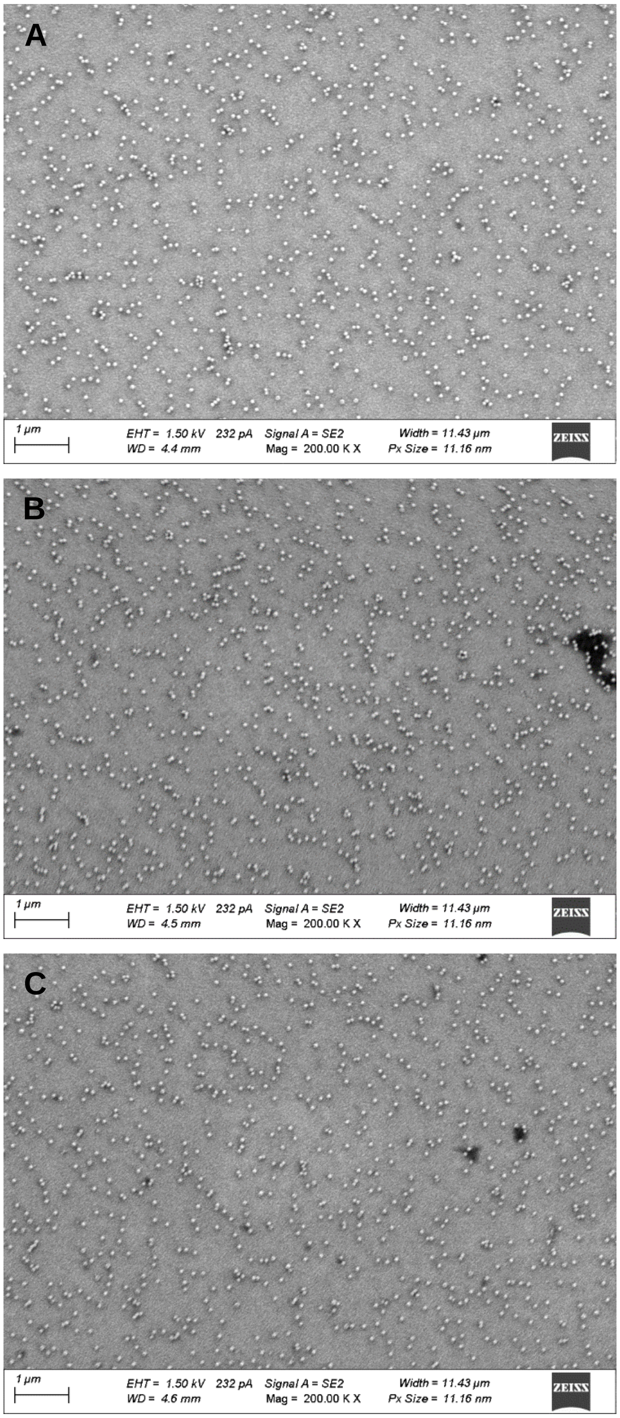

Figure S2. Representative SEM images for the assembly of (A) AuFilm-IgG + AuNP-NBT/Ab, (B) AuFilm-NBT/IgG + AuNP-Ab, and (C) AuFilm-NBT/IgG + AuNP-NBT/Ab.

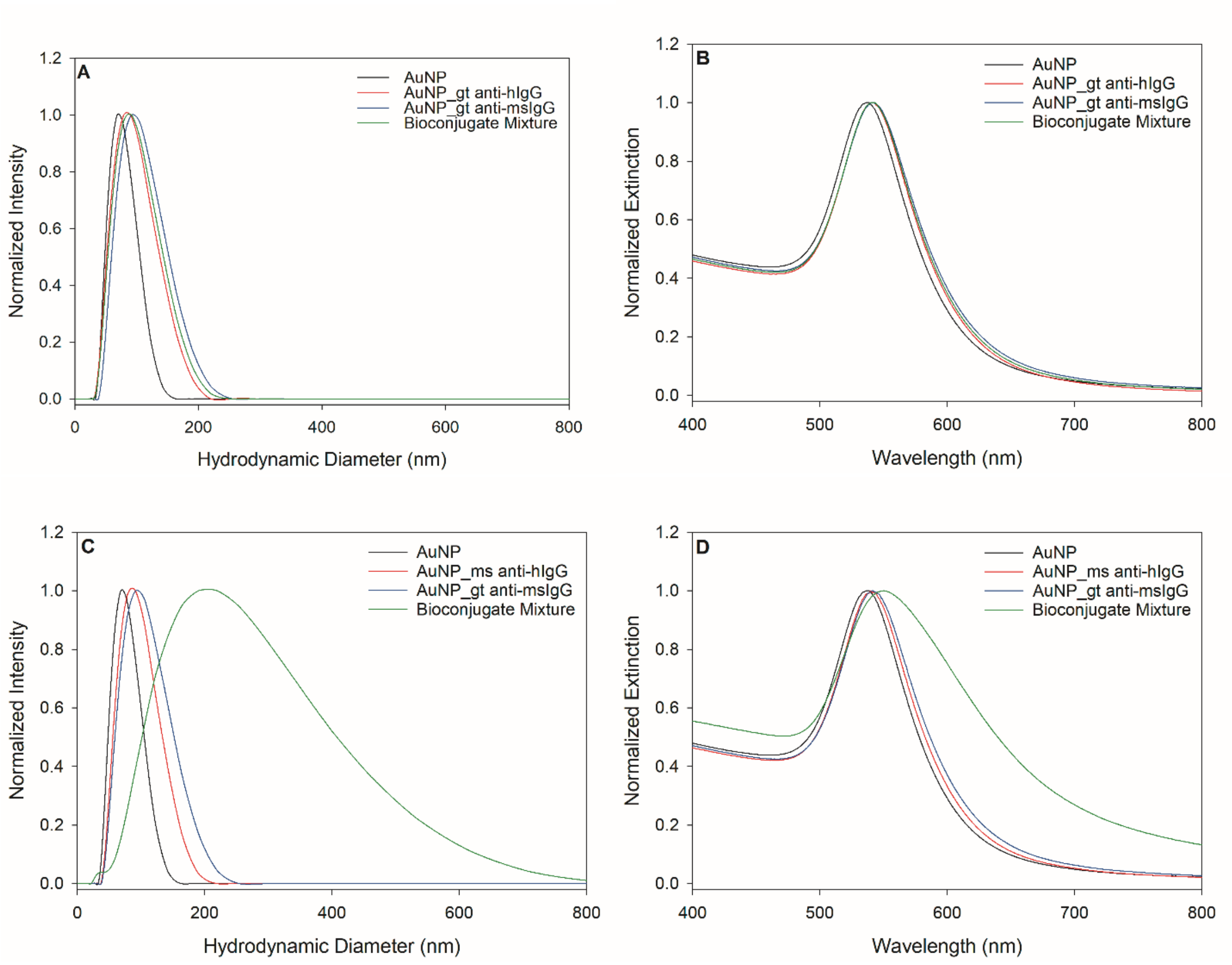

Figure S3. (A) DLS derived hydrodynamic diameter and (B) extinction spectra of AuNPs, AuNP-goat anti-human bioconjugate, AuNP-goat anti-mouse bioconjugate, and a mixture of both. (C) DLS derived hydrodynamic diameter and (D) extinction spectra of AuNPs, antibody-modified AuNPs, and the mixture of AuNP-mouse anti-human and AuNP-goat anti-mouse IgG bioconjugates.

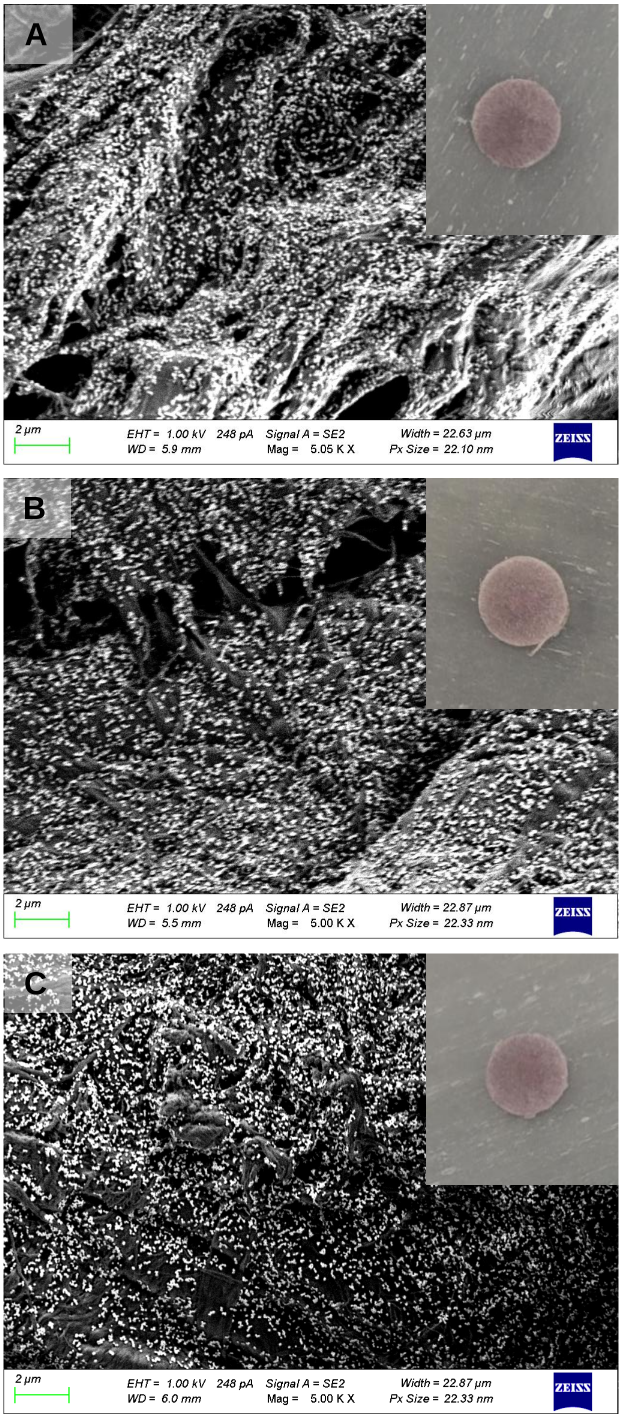

Figure S4. Digital photos and SEM images for (A) as prepared capture substrate (AuNP-IgG), (B) after binding the detection probes (AuNP-NBT/Ab) on A, (C) after applying enhancement probes (AuNP-IgG) on B.

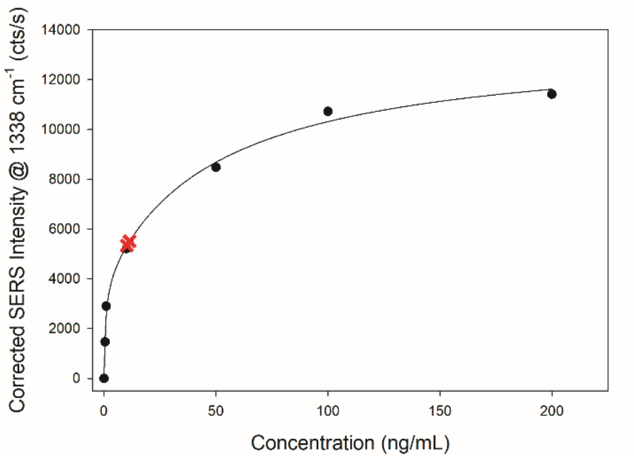

Figure S5. Calibration curve as a function of hIgG concentration obtained under the optimized parameters via three-layered sandwich VFIA. Two human serum samples were diluted 1:10^6 in PBS and analyzed (red x).

References

1. Kimling, J.; Maier, M.; Okenve, B.; Kotaidis, V.; Ballot, H.; Plech, A., Turkevich Method for Gold Nanoparticle Synthesis Revisited. *J. Phys. Chem. B* **2006**, *110*, 15700-15707.
2. Magura, J.; Zeleňáková, A.; Zeleňák, V.; Kaňuchová, M., Thiol-Modified Gold Nanoparticles Deposited on Silica Support Using Dip Coating. *Appl. Surf. Sci.* **2014**, *315*, 392-399.
3. Huang, C.-C.; Cheng, C.-Y.; Lai, Y.-S., Paper-Based Flexible Surface Enhanced Raman Scattering Platforms and Their Applications to Food Safety. *Trends Food Sci. Technol.* **2020**, *100*, 349-358.
4. Lartey, J. A.; Harms, J. P.; Frimpong, R.; Mulligan, C. C.; Driskell, J. D.; Kim, J.-H., Sandwiching Analytes with Structurally Diverse Plasmonic Nanoparticles on Paper Substrates for Surface Enhanced Raman Spectroscopy. *RSC Adv.* **2019**, *9*, 32535-32543.
5. Yu, W. W.; White, I. M., Inkjet Printed Surface Enhanced Raman Spectroscopy Array on Cellulose Paper. *Anal. Chem.* **2010**, *82*, 9626-9630.
6. Frimpong, R.; Jang, W.; Kim, J.-H.; Driskell, J. D., Rapid Vertical Flow Immunoassay on AuNP Plasmonic Paper for Sers-Based Point of Need Diagnostics. *Talanta* **2021**, *223*, 121739.
7. Lopez, A.; Lovato, F.; Hwan Oh, S.; Lai, Y. H.; Filbrun, S.; Driskell, E. A.; Driskell, J. D., Sers Immunoassay Based on the Capture and Concentration of Antigen-Assembled Gold Nanoparticles. *Talanta* **2016**, *146*, 388-393.
